# To be or not to be born at the right time: lessons from ice hockey

**DOI:** 10.3389/fspor.2024.1440029

**Published:** 2024-07-10

**Authors:** Simon Grondin

**Affiliations:** École de Psychologie, Université Laval, Québec, QC, Canada

**Keywords:** relative age effect, ice hockey, talent identification, birthdate effect, categories in sport, age discrimination, personal development

Perhaps surprisingly, an individual's date of birth has a notable and long-term influence on their development. Any kindergarten or grade 1 teacher will speak of the remarkable difference, physically and/or psychologically, between the younger and older children in their class. While this influence had been documented in schools since the 1960s (1), it was not until the 1980s that the phenomenon was noted in sport, in ice hockey in particular (2, 3). Curiously enough, in sport, this seemingly-important difference has been long disregarded. However, despite over 40 years of research on this issue, the problems it causes remain unresolved.

The effect of an individual's birthdate on sport participation and attainment, eventually referred to as relative age effect (RAE), went from an object of curiosity to a cruel and pervasive reality that is a worldwide and widespread phenomenon (4–6). Consistently, it is a key factor explaining success in sport, and in talent identification selections. It eventually became popular as an example for explaining that success in general, in several spheres of activity, could be related to an arbitrary decision of how and when to group individuals to provide consistency in instruction and training.

## The 1984 paper

As a child born at the end of November and playing ice hockey, I suspected that there was something wrong, unfair, with the categorization system. Indeed, I realized it more clearly as a baseball^1^ player in Little League: instead of the disadvantage caused by a cutoff date set on January 1st, I experienced some advantage of a system based on chronological age with a cutoff date set on August 1st. I certainly did not know how to translate this curiosity into a scientific question, but I guess the issue was sleeping inside of the teenager I was who eventually undertook university studies to become a physical educator.

The 1984 article on the RAE in ice hockey (Grondin et al.) was based on my Master's thesis, defended in October 1982 in the *Département de kinanthropologie* at *Université de Sherbrooke* (8). The thesis was supervised by Dr. Paul Deshaies. The content of the thesis was first presented at the 51st Annual meeting of the “*Association canadienne-française pour l*’*avancement des sciences*” (ACFAS) held in Trois-Rivières in May 1983. The abstracts of two talks were published in the proceedings of the meeting. One talk presented the research question and the data showing that there was a real problem (9); and the second talk was dedicated to a suggestion, based on birthdate, to solve the problem (10). It was only in 1993 that I met Roger Barnsley and Gus Thompson, when they organized a symposium on the RAE during the annual meeting of the *Canadian Psychological Association* held that year in Montréal. They were kind enough to invite me to participate in the symposium, lending me the podium to talk about the RAE in ice hockey (11) while they were talking about this effect on academic achievement (12), mental health (13), and sport in general (14).

It was in 1984 that an article based on the thesis was published. The data, which were collected at the *Fédération Québécoise de hockey sur glace* (now Hockey Québec) and *Fédération de volley-ball du Québec*, revealed a series of critical messages:

(1) In competitive ice hockey (*n* = 3,826), many more players were born in the first quarter of the year than in the fourth. This effect was present and strong in all competitive minor ice hockey categories, beginning with Atom (9- and 10-year-old players) up to Junior ice hockey (under 20). This effect was not as strong but statistically significant in the National Hockey League.
(2) Within each competitive minor ice hockey category, the effect was stronger in higher classes of competition (AA > BB > CC)^2^, that is, in cities with a larger population. For example, amongst Pee Wee AA players (11–12 years of age), 47.8% and 8.9% were born in the 1st and 4th trimester, respectively. All this to say that when there are more players competing for a position on a team, the effect of birthdate is stronger.
(3) In general, there was no effect of birthdate in volleyball, for either female and male players, probably because there was less competition for obtaining a position on a team, this sport being less popular than ice hockey in Canada (and maybe because at the time, teams were formed in schools, which was not the case in ice hockey). Nevertheless, there was a birthdate effect for regional volleyball elite teams involving 14- and 15-year-old male players, teams that were built for a provincial competition. In other words, more competition for obtaining a place on the team resulted in an effect; therefore, the effect observed in ice hockey was not due the fact that this sport had physical contact (or some violence) since the effect also seemed to occur in volleyball when certain conditions are met.
(4) The final main message of the paper was a recommendation to improve the categorization system, even based on chronological age. The key point involved recognizing that the problem was caused by the adoption of a “yearly-based system” instead of a “monthly-based system” that would not be a multiple of 12. Two examples were given in the article, with 15- and with 21-month long categories. In the latter case, in a four-category system, the curriculum would last 7 years, each player going through the 7 years would play 2 years in 3 categories, and 1 in another category. This single season in a category would occur at a moment of the curriculum (category 1, 2, 3, or 4) that would depend on the system adopted and the birthdate of a player.

The 1982 thesis, but not the 1984 paper, also contained a non-trivial recommendation: if the problem cannot be solved within a singular sport, we should at least avoid adopting the same cutoff date in all sports. This suggestion went squarely in the opposite direction of a 1980 Québec government report on age categories in sport (15). Since, there have been several other recommendations [see (16)], including one where it is suggested to have two ice hockey seasons (September–December and January–April) per year, each half-season having different cutoff dates, for instance, January 1st and July 1st (17).

## Other ice hockey data, since

Since the publication of Grondin et al. (3) and Barnsley et al. (2), there have been several papers on birthdate effects in ice hockey [e.g., (18–25)]. Some recent papers were dedicated to Russian or Czech ice hockey [e.g., (26, 27)], or to women's ice hockey [e.g., (28, 29)]. Other research has established that there is not much difference on anthropometric measures of ice hockey players born in the different trimesters of the year (30, 31). Finally, several papers were dedicated to the case of the NHL, including the fact that the effect is weaker there than in minor ice hockey, a finding referred to as a reversal effect (18, 32–35).

Amongst the multiple articles on birthdate's effect on participation in ice hockey, there is one less cited paper that contains quite precious data (36). The data are interesting because it shows clearly, and how rapidly, the effect is installed. In Québec, the cutoff date was January 1st until the 2002–2003 season. For six ice hockey seasons, from 2002–2003 to 2007–2008, the cutoff date was October 1st, this date being the cutoff for schools in Québec. After the 2007–2008 season, the cutoff date for ice hockey was moved back to January 1st.

According to Table 2 of Lavoie et al. (36), for the highest competitive level of 15- and 16-year-old players (Midget AAA), 44.94% were born in October, November, or December during the 2007–2008 season. The following season, when the cut-off date went back to January 1st, the percentage for this trimester was 19.58%; and, for the 2009–2010 season, went down to 11.74%. In 2007–2008. 23.97% of players were born in January, February, or March, then the second most advantageous trimester. Two years later, 41.30% were born in the first trimester. Not only does this manipulation of the cutoff date show the cause to effect relationship between the birthdate and participation in elite ice hockey, but this effect happens extremely rapidly. Similar effects have been noted by Helsen et al. (37) in elite soccer players.

While the administrative mechanisms driving birthdate effects seem simple, the psychological factors underpinning the effect are complicated. In a recent article about talent identification in ice hockey, Fortin-Guichard et al. (38) showed the importance of self-regulation planning and gaze behaviour for identifying talent of 15–16-year-old players. What is interesting in this paper is the uniqueness of the method used: (1) only players selected after the first two rounds of selection (“sleepers”) were under investigation and (2) scouts were asked, 3 years after the draft (players now competing at the Junior level) to identify who they would have liked to pick, out of players selected in the first two rounds. Out of the 70 remaining players who were tested as a Midget, 15 were identified by scouts. Only 7 out of the 44 players born from January to June were selected, while 8 out of 26 players were born from July to December. Note that, amongst the players tested and selected in the first two rounds, 21 out of 25 players were born from January to June. In other words, scouts do not compensate for the enormous bias caused by the moment of birth and the categorization system.

## Just to make it clear

**Figure 1:**
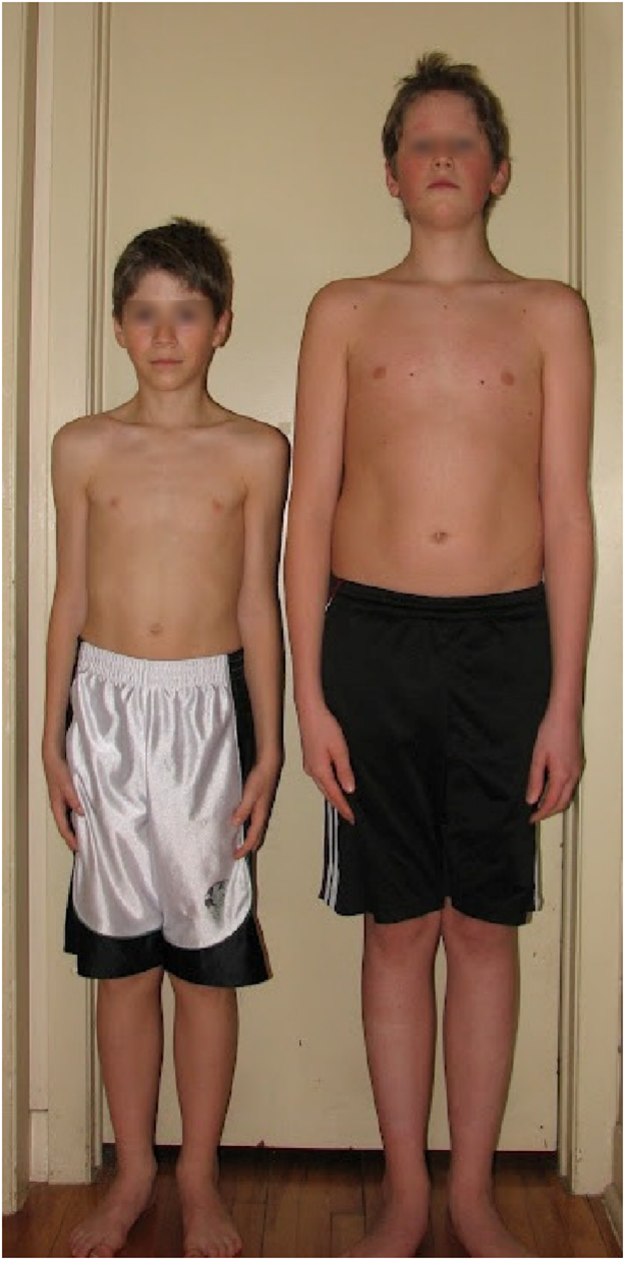
The taller boy on the right is 1 month younger that the smaller one on the left, but because one was born in January and the other in December, the taller boy will stay in the same age category the following season and the smaller one will move into a category with older children (photo from (17); with permission of the persons, now adults, on the photo).

There is a clear and long history of evidence regarding the heterogeneity of development. An old article by Bouchard and Roy (39) revealed that, amongst participants in the famous Québec Pee Wee tournament (for players 11 and 12 years of age), players' “bone age” (an indicator of skeletal maturity) varied from 7 to 14 years. But because a picture is worth a thousand words, let's use a photo to make the point clear about this potential heterogeneity. The two children in Figure 1 played soccer and went to school together. Their age? About the same. Despite their physical difference, there is only 1-month difference between them in chronological age. The fun fact here is this one: the taller one was born in January, and the other a bit earlier, in December. In other words, the following season, the taller one had to remain in the same category, and the smaller one had to move into a category with older children.

The problem can also be considered another way. To keep it simple, let's consider two children, Player A born on January 1st and Player B born on December 31st of the same year. There is a high risk that a coach not sensitive to the impact of birthdate, at the moment of building a team, will make decisions based on a spontaneous dichotomy, “first year player” vs. “second year player”. If the objective of the coach is to win, the older player has a better chance to be part of the team. But if the objective is to detect talent, and eventually contribute to its development, two questions should be asked. (1) A year from now, will Player B be as good as, or better than, Player A is now? (2) Will the difference between current player A and player B in two years be smaller or larger than the current difference between players A and B?

## A final word

The past 40 years have revealed something remarkable and deplorable: even when a problem is well-known, obvious, and well understood, even when there are potential solutions, it remains extremely difficult to activate changes in a system! It is difficult to know with certainty whether the discrimination caused by birthdate would be reduced, but relatively simple solutions are worth testing, be it the adoption of 15- or 21-month age categories, or at least, with a categorisation avoiding multiples of 12 months; or by forcing to have at least 3 players per trimester in competitive teams.

To close this article, let's use a translation of the last sentence of the 1984 paper, and of the 1982 thesis, that was a message for any coach or educator [see also (40)]: “In brief, it must be remembered that two children born in the same year do not necessarily have the same age.” Even after 40 + years, it seems many in the sport system have still not gotten this message.

## Footnotes

^1^There is also an effect of the moment of birth in baseball, and this effect is important in Japan where the cutoff date is April 1st (7).

^2^There were AA teams in cities with more than 45,000 people, and BB teams in cities with 20,001–45,000 people.
